# Chemical profiling and *in silico* evaluation of a *Matricaria chamomilla* L. extract: exploring its antioxidant, antibacterial, antidiabetic, anti-inflammatory, and anticholinergic activities

**DOI:** 10.3389/fnut.2026.1852820

**Published:** 2026-06-18

**Authors:** Abderrazek Ferhat, Mohammed Messaoudi, Khalil Guelifet, Ilham Ben Amor, Wafa Zahnit, Khadra Afaf Bendrihem, Azzeddine Zeraib, Hamza Ouakouak, Mokhtar Benmohamed, Mohamed Amine Ferhat, Imane Larkem, Huda Alsaeedi, Aftab Aslam Parwaz Khan, Mikhael Bechelany, Furong Tian, Ahmed Barhoum

**Affiliations:** 1 Laboratory of Research in Science and Environment: Bioresources, Geochemistry-Physics, Legislation and Socio-Economic Development, Faculty of Sciences and Technology, University of Tamanghasset, Tamanrasset, Algeria; 2 Laboratory of Research on Bio-Active Products and Valorization of Biomass, Ecole Normale Supérieure, Algiers, Algeria; 3 Department of Process Engineering and Petrochemical, Faculty of Technology, University of El Oued, El Oued, Algeria; 4 Department of Chemistry, Faculty of Sciences, University of Ferhat ABBAS Setif 1, Setif, Algeria; 5 Biotechnology, Water, Environment and Health Laboratory, Faculty of Natural and Life Sciences, University of Abbes Laghrour, Khenchela, Algeria; 6 Laboratory of Genetics, Biotechnology and Valorization of Bio-Resources, University of Biskra, Biskra, Algeria; 7 Department of Physics, Saida Higher Teacher Training School, Saida, Algeria; 8 Laboratory N—Body and Structure of Matter, Department of Chemistry, Higher Normal School of Kouba (ENS), Algiers, Algeria; 9 FSNV, Département Sciences Agronomiques, Université Ferhat Abbas de Sétif, Sétif, Algeria; 10 Laboratoire d'amélioration et développement de la production végétale et animale (LADPVA), Sétif, Algeria; 11 Department of Chemistry, College of Science, King Saud University, Riyadh, Saudi Arabia; 12 Center of Excellence for Advanced Materials Research, King Abdulaziz University, Jeddah, Saudi Arabia; 13 Institut Européen des Membranes, IEM, UMR 5635, Univ Montpellier, ENSCM, CNRS, Montpellier, France; 14 Nanolab Research Centre, Physical to Life Sciences Research Hub, Technological University Dublin, Dublin, Ireland; 15 School of Chemical and BioPharmaceutical Sciences, Technological University Dublin, Dublin, Ireland

**Keywords:** anti-Alzheimer, antibacterial, antidiabetic, anti-inflammatory, antioxidant, *Matricaria chamomilla* L., medicinal plants

## Abstract

**Introduction:**

This study aimed to investigate the phytochemical composition and multifunctional therapeutic potential of a hydroethanolic extract of *Matricaria chamomilla L*. collected from the semi-arid region of Algeria. The research focused on evaluating its antioxidant, antimicrobial, antidiabetic, anti-inflammatory, neuroprotective, and potential anticancer activities.

**Methods:**

Total polyphenol and flavonoid contents were determined using spectrophotometric methods. The phytochemical profile was characterized by Liquid Chromatography–Tandem Mass Spectrometry (LC-MS/MS). Antioxidant activity was assessed using DPPH and galvinoxyl radical scavenging assays. Antimicrobial activity was evaluated against bacterial and fungal strains using inhibition zone measurements. Enzyme inhibitory activities against α-amylase, acetylcholinesterase, and butyrylcholinesterase were investigated *in vitro*. Anti-inflammatory activity was evaluated through protein denaturation inhibition and carrageenan-induced paw edema assays. Molecular docking studies were performed to assess the interactions of identified compounds with breast cancer targets ERα and HER2.

**Results:**

The extract contained high levels of total polyphenols (41.9 mg gallic acid equivalent/g extract) and flavonoids (17.1 mg rutin equivalent/g extract). LC-MS/MS analysis identified several bioactive compounds, including caffeic acid, gallocatechin gallate, and apigenin-7-O-glucoside. The extract exhibited moderate antioxidant activity, with IC50 values of 324.4 μg/mL and 22.7 μg/mL for DPPH and galvinoxyl assays, respectively. Strong antimicrobial effects were observed against *Staphylococcus aureus* (22 mm inhibition zone) and Candida albicans (18 mm inhibition zone). The extract also demonstrated potent inhibitory activities against α-amylase (IC_50_ = 440.6 μg/mL), acetylcholinesterase (IC_50_ = 3.11 μg/mL), and butyrylcholinesterase (IC_50_ = 28.7 μg/mL), surpassing standard inhibitors. In vitro anti-inflammatory activity reached 98.4% inhibition of protein denaturation at 2000 μg/mL, while in vivo edema inhibition reached 38.81%. Molecular docking revealed strong binding affinities of α-tocopherol and apigenin-7-O-glucoside toward ERα and HER2 receptors.

**Discussion:**

The findings demonstrate that *Matricaria chamomilla L*. possesses significant pharmacological potential due to its rich phytochemical composition and broad-spectrum biological activities. The observed antioxidant, antimicrobial, enzyme inhibitory, anti-inflammatory, and molecular docking results suggest that this plant could serve as a promising natural source for the development of therapeutic agents targeting diabetes, neurodegenerative disorders, microbial infections, and breast cancer. Further pharmacological and clinical investigations are warranted to validate these bioactivities and support future drug development applications.

## Graphical Abstract

**Figure:**
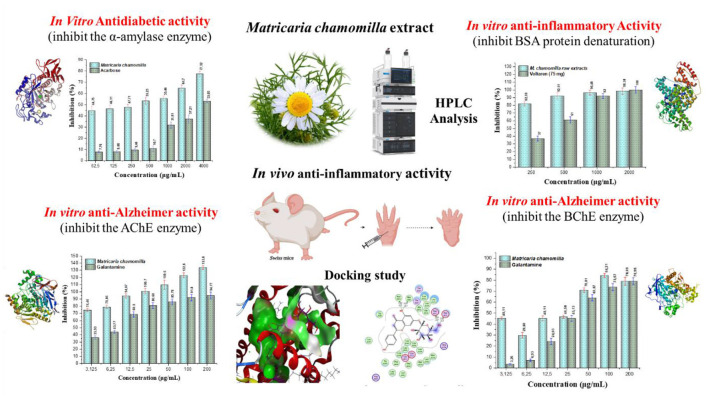

## Highlights

- *Matricaria chamomilla L*. extract contains high levels of phenolics and flavonoids that contribute to its bioactive properties.
- The extract shows potent antioxidant, antidiabetic, and anticholinergic enzyme activities.
- It exhibits strong antibacterial and antifungal activity against *Staphylococcus aureus* and *Candida albicans*.
- The extract has significant anti-inflammatory effects on *in vitro* and *in vivo* models.

## 1 Introduction

Medicinal plants have contributed to human health for millennia by providing remedies for many conditions, ranging from digestive disorders to chronic diseases (1, 2). Their bioactive compounds (e.g., alkaloids, flavonoids, terpenes, and saponins) are now increasingly tested also in modern pharmacology (3, 4), to exploit their various therapeutic activities (e.g., antioxidant, antimicrobial, antidiabetic effects). Their application spans various fields, including pharmaceuticals, cosmetics, and nutrition, demonstrating their broad utility and importance in health management. Ongoing research continues to explore and validate the plant health benefits, contributing to a deeper understanding of their potential therapeutic applications (5–7).

The therapeutic properties of *Matricaria chamomilla* L., commonly known as chamomile, have been known for centuries (8–10). This plant, part of the *Asteraceae* family, has distinctive aromatic qualities and contains many secondary metabolites, including flavonoids, terpenes and sesquiterpenes (11). Chamomile extracts are particularly rich in phenolic compounds, flavonoids such as apigenin and quercetin, and essential oils containing chamazulene and α-bisabolol that contribute to their bioactive effects, particularly to their antimicrobial and antioxidant properties. Phytochemical analyses have identified key components, such as chamazulene and apigenin, that contribute to the plant therapeutic benefits (12, 13). Environmental factors, including soil composition, climate conditions and geographical location, significantly influence the phytochemical profile of medicinal plants, suggesting that chamomile from different regions may possess distinct therapeutic properties (11, 14). The therapeutic applications of *M. chamomilla* L. span from traditional remedies to clinical applications (15). Chamomile has historically been used for gastrointestinal problems, insomnia, and inflammation, due to its antispasmodic, anxiolytic and anti-inflammatory properties (16, 17). In modern medicine, its potential is being explored for the management of chronic conditions, including diabetes and neurodegenerative disorders, due to its ability to modulate blood glucose levels and offer neuroprotection. Several studies have demonstrated the efficacy of chamomile in neutralizing free radicals, inhibiting pathogen growth, suppressing α-amylase activity, and reducing inflammation. These findings underscore its therapeutic potential and its importance as a natural remedy for future health applications (11, 18).

Recent studies using inductively coupled plasma-optical emission spectrometry have detected only trace amounts of toxic elements in Algerian *M. chamomilla* L. (9, 19). However, the full range of pharmacological activities of *M. chamomilla* L. from semi-arid regions has not been explored yet. To close this knowledge gap, we performed a comprehensive, multi-target evaluation of a hydromethanolic *M. chamomilla* L. extract from Algeria using four complementary approaches: (i) high-performance liquid chromatography-ultraviolet spectroscopy (HPLC-UV) profiling to determine and quantify its major phytoconstituents; (ii) *in silico* molecular docking to predict how these compounds bind to key therapeutic targets, providing mechanistic hypotheses at the atomic level; (iii) *in vitro* bioassays (cell-based and enzyme inhibition tests) to measure the extract antioxidant, antibacterial, antidiabetic, anticholinergic and anti-inflammatory effects; and (iv) *in vivo* carrageenan-induced paw edema mouse model to validate the extract anti-inflammatory activity. The novelty of our methodology lies in this integrated pipeline that for the first time, links detailed chemical characterization, computational target prediction, and cellular and animal-based bioactivity assays in a single study. This synergy between analytical chemistry, computer modeling and experimental pharmacology strengthens the robustness of our findings and also streamlines the identification of lead candidates for drug development.

## 2 Experimental

### 2.1 Reagents and materials

Total polyphenolic content (TPC) was assessed using the Folin–Ciocalteu reagent, which consists of phosphomolybdic and phosphotungstic acids. Sodium carbonate was employed to provide the alkaline medium necessary for the activation of the Folin-Ciocalteu reagent. Antioxidant activity was evaluated using DPPH, ABTS, and galvinoxyl assays. α-Tocopherol (vitamin E), BHT, BHA, and quercetin were used as reference compounds. Gallic acid was used as a phenolic standard, while aluminum chloride and potassium acetate were employed for flavonoid quantification. For antibacterial and antidiabetic assays, starch, α-amylase, hydrochloric acid, and potassium iodide were used. Anticholinesterase activity was evaluated using acetylthiocholine iodide, butyrylthiocholine chloride, and DTNB. Bovine serum albumin, Tris-HCl buffer, and carrageenan were used for anti-inflammatory activity assessment. All reagents and solvents were of analytical grade and purchased from Sigma-Aldrich (Germany). As well as, antimicrobial activity was tested against reference strains, including *Staphylococcus aureus* (ATCC 6538), *Bacillus subtilis* (ATCC 6633), *Pseudomonas aeruginosa* (ATCC 9027), *Escherichia coli* (ATCC 8739), and *Candida albicans* (ATCC 10231).

### 2.2 *M. chamomilla* L. sampling and preparation

In June 2020, aerial parts of *M. chamomilla L*. were harvested at the El-Guetfa region of M'sila, a semi-arid area in Algeria (coordinates: 35°44'26"N, 3°23'05"E). The plant was subsequently identified by experts at the University of El Oued, Algeria. The plant materials were immediately transported to the laboratory in clean, airtight containers to minimize degradation or contamination. Once in the laboratory, samples were washed with deionized water more than three times to remove dirt and impurities. After washing, the samples were spread in a single layer on clean trays and subjected to controlled shade-drying at 25 °C for 2 weeks until a constant weight was achieved. This passive dehydration was conducted in a well-ventilated laboratory environment, away from direct sunlight, to prevent the photodegradation and thermal decomposition of sensitive thermolabile compounds. This non-accelerated drying approach is consistent with established protocols for preserving the phytochemical profile of medicinal plants (50, 51). The dried plant material was finely ground using an agate mortar and pestle to achieve a particle size < 200 μm, ensuring uniformity. The resulting powdered samples were stored in airtight containers in a cool, dry environment at 4 °C to maintain their integrity.

### 2.3 Phytochemical extraction

Phenolic compounds were extracted from the *M. chamomilla* L. powder using a methanol-based solvent system to facilitate the extraction of bioactive compounds for biological tests. Fifteen g of the dried plant powder was macerated in 500 mL of a methanol/water mixture (8:2, v/v) at 25 °C for 24 h. Continuous mechanical stirring was applied to enhance the solvent penetration and extraction efficiency. After 24 h, the mixture was filtered to separate the liquid extract from the plant residue. Maceration and filtration were repeated three times to ensure thorough extraction of phenolic compounds from the plant material. The collected filtrates were pooled and concentrated with a rotary evaporator (Buchi R-200, Switzerland) at 40 °C under decreased pressure to eliminate the solvent. The resulting crude extract was stored at −4 °C to ensure its stability. The extraction yield was determined using Equation 1:

$$\text{Yield}(\%)\text{=}\frac{\text{Weight of dried crude extract }(\text{g})}{\text{Weight of dried plant material }(\text{g})}\;\times \text{ 100}$$ (1)

### 2.4 Total polyphenol content (TPC)

The TPC was quantitatively determined according to the Singleton–Rossi method employing the Folin–Ciocalteu reagent, a well-established and extensively validated protocol in phytochemical investigations. The assay is based on a coupled electron-transfer mechanism, whereby phenolic hydroxyl groups reduce the heteropoly acids present in the reagent, leading to the formation of a stable blue chromophore. The intensity of this chromogenic response is directly correlated with the total reducing capacity of phenolic constituents. Spectrophotometric quantification was performed by monitoring the absorbance in the range of 750–765 nm, corresponding to the maximum absorption of the formed complex. For the assay, 20 μL of *M. chamomilla* L. extract was mixed with 100 μL of 1:10 diluted Folin-Ciocalteu reagent and 75 μL of 7.5 wt %. The inorganic salt sodium carbonate. The mixture was incubated in the dark at 25 °C for 2 h. Absorbance was measured at 765 nm with a UV-Vis spectrophotometer (LAMBDA™ 365, PerkinElmer, USA). TPC was quantified as μg gallic acid equivalent (GAE)/mg extract using a gallic acid calibration curve.

### 2.5 Total flavonoid content (TFC)

TFC was determined by creating a compound between flavonoids and Al^3+^ ions, following the modified Shraim method (20). In a 96-well microplate, 50 μL of *M. chamomilla* L. extract was mixed with 10 μL Potassium acetate, 130 μL of methanol, and 10 μL Aluminum nitrate and incubated in the dark for 40 min. The absorbance was measured at 415 nm with a UV-Vis spectrophotometer (UV-2450, Shimadzu, Duisburg, Germany). TFC was determined based on a quercetin calibration curve (0–40 μg/mL) and quantified as milligrams of quercetin equivalents per gram of crude extract (mg QE/g extract).

### 2.6 Characterization by LC-MS/MS analysis

The phenolic chemical profile of *Matricaria chamomilla* L was examined utilizing an LC-MS/MS approach with a UPLC-ESI MS triple quadrupole Shimadzu 8040 system. Chromatography was performed on a 150 × 3 mm, 3 μm, RP-18 column using an isocratic phase, a total flow of 200 μL/min, and 2 μL sample injections. A gradient of 30% water, 0.1% formic acid, and 70% methanol were employed as the mobile phase. The electrospray ionization (ESI) source of the mass spectrometer was operated in negative ionization scan mode. The identification of compounds was achieved through comparison of their mass spectra with literature data based on their *m*/*z* values. The MS/MS employs the following ESI conditions: 3.00 L/min nebulizing gas flow, 400 °C heat block, 15.00 L/min drying gas flow, 350 °C interface temperature, 250 °C DL temperature, 230 KPs CID gas, and 6.00 Kv conversion dynode.

### 2.7 *In vitro* antioxidant activity

The antioxidant activity of the extract was ascertained using DPPH (21), ABTS (22), β-carotene (23), and GOR radical scavenging assays (24). For the DPPH assay, 1 mL of 0.1 mM DPPH solution was combined with 40 μL of extract and incubated in the absence of light for 30 min. For the ABTS assay, 40 μL of extract was mixed with 1 mL of 7 mM ABTS solution and incubated for 16 h. In the β-carotene assay, 40 μL of extract was combined with 1 mL of β-carotene emulsion and incubated for 30 min. For the GOR assay, 40 μL of extract was mixed with 1 mL of GOR solution and incubated for 30 min. Absorbance was determined at 517 nm (DPPH), 734 nm (ABTS), 470 nm (β-carotene), and 428 nm (GOR) with a UV-Vis spectrophotometer. The BHT assay was performed with BHT concentrations of 10, 20, 50, 100, and 200 μg/mL mixed with 1 mL of DPPH solution. After incubation for 30 min, absorbance was measured at 517 nm. The % inhibition for all experiments was computed using Equation 2:

$$\text{I}(\%)\text{=}\frac{\left({\text{A}}_{\text{0}}\;{\text{A}}_{\text{1}}\right)}{{\text{A}}_{\text{0}}}\;\times \text{ 100}$$ (2)

where *A*_0_ is the absorbance of the control (DPPH, GOR, β-carotene, ABTS, or BHT solution) and *A*_1_ is the absorbance of the tested material. Data are presented as IC_50_ and *A*_0.5_, denoting the concentration required to block 50% of free radicals.

### 2.8 Antibacterial activity *in vitro*

The antibacterial activity of the *M. chamomilla* L. extract was evaluated with the disk diffusion technique against Gram-negative strains (*P. aeruginosa* and *E. coli*), Gram-positive strains (*B. subtilis* and *S. aureus*), and the fungal strain *C. albicans*. Paper disks (diameter = 6 mm) were impregnated with 35 μg of the extract solution and placed on agar plates previously inoculated with microorganisms. For comparison, reference antibiotics (carbenicillin, fosfomycin, erythromycin, and cephalexin; 35 μg per disk) were employed for Gram-positive strains, and fosfomycin for Gram-negative strains. Antibacterial activity was assessed by measuring the inhibition zones surrounding the disks after incubation at 37 °C for 2 h.

To determine the Minimum Inhibitory Concentration (MIC), a serial dilution technique was employed with extract concentrations ranging from 1 to 64 μg/mL in nutrient broth that was inoculated with microbial suspensions and incubated at 37 °C for 24 h. The tested strains included *B. subtilis, Listeria monocytogenes* CIP82110, *S. aureus* (Gram-positive bacteria); *Klebsiella pneumoniae* CIP 8291, *P. aeruginosa, E. coli* (Gram-negative bacteria); and *Mucor ramannianus, Aspergillus flavus, Penicillium expansum, Fusarium culmorum* (fungi). The MIC was defined as the minimal concentration of the extract that prevented observable microbiological growth, determined by observing the turbidity in the broth cultures compared with the control culture.

### 2.9 *In vitro* antidiabetic activity

The antidiabetic potential of the *M. chamomilla* L. extract was assessed with the α-amylase inhibition assay (25). In this procedure, 50 μL of α-amylase solution (1 U) was combined with 25 μL of the extract at several concentrations in a 96-well microplate and incubated at 37 °C for 10 min. Next, 50 μL of a 0.1 wt % starch solution was added, followed by another 10-min incubation at 37 °C. Then, 25 μL of 1 M HCl (prepared by diluting 4.17 mL of concentrated HCl in 45.83 mL H_2_O) and 100 μL of IKI solution were introduced. Absorbance was then measured at 630 nm with a UV-Vis spectrophotometer. Acarbose served as reference. The proportion of α-amylase inhibition was determined using Equation 3:

$$I(\%)=\frac{\text{1-}[\left(\text{Ac-Ae}\right)\text{- }\left(\text{As-Ab}\right)]}{\left(\text{Ac-Ae}\right)}\;\times \text{ 100}$$ (3)

Where, Ac: absorbance of starch, HCl, IKI, enzyme buffer, and extract solvent, Ae: absorbance of starch, enzyme, HCl, IKI, and extract solvent, As: absorbance of extract, enzyme, starch, HCl, and IKI, and Ab: absorbance of extract, buffer, and IKI.

### 2.10 Anticholinesterase activity *in vitro*

The anticholinesterase activity of the *M. chamomilla L*. extract was evaluated by its ability to inhibit butyrylcholinesterase (BChE, from horse serum) and acetylcholinesterase (AChE, from electric eel) using a modified method (26). BuCl and ACI were used as substrates, and DTNB to detect the cholinesterase activity. In a 96-well plate, 150 μL of 100 mM sodium phosphate buffer (pH 8) was mixed with 10 μL of extract (4 mg/mL in methanol) at various concentrations and 20 μL of AChE or BChE. After 15 min at 25 °C, 10 μL of DTNB (0.5 mM) and 10 μL of BuCl (0.2 mM) or of ACI (0.71 mM) were added to start the reaction. The resulting yellow 5-thio-2-nitrobenzoate anion reacted with DTNB, and absorbance was measured at 412 nm with a UV-Vis spectrophotometer. Galantamine was used as reference. The inhibition percentages at 25, 50, 100, and 200 μg/mL extract were used to calculate the IC_50_ values (Equation 4).

$$\begin{array}{l} I\left(\%\right)=\frac{E\;-\;S}{E}\;\times \;100 \end{array}$$ (4)

Where, *E* is the enzymatic activity without the extract (negative control) and *S* is the enzymatic activity in the presence of the extract (sample).

### 2.11 Acute toxicity

An acute oral toxicity study was conducted to determine the safety profile of *Matricaria chamomilla*, following OECD Guideline 423 (52). Groups of fasted female mice (*n* = 5 per group) received a single oral administration of the extract at incremental dosages of 275, 1,562, and 2,000 mg/kg. Following administration, the animals were meticulously monitored during the initial hours and subsequently observed daily for 14 days to identify any signs of toxicity, including behavioral changes, clinical symptoms, or mortality. This assessment facilitated the calculation of the median lethal dose (LD_50_) and supplied critical toxicological information to warrant additional pharmacological research on the extract.

### 2.12 Anti-inflammatory activity *in vitro*

The anti-inflammatory potential of the *M. chamomilla L*. extract was evaluated by its ability to inhibit BSA denaturation induced by heat (72 °C), following a modified method described by Kandikattu et al. (27). In this assay, 1 mL of extract or diclofenac sodium was mixed with 1 mL of 0.2% BSA in Tris-HCl buffer (pH 6.6). The mixture was incubated at 37 °C for 15 min, heated to 72 °C for 5 min, and then cooled to 50 °C. The turbidity was measured at 660 nm with a UV-Vis spectrophotometer (UV-2450, Shimadzu, Duisburg, Germany). A blank was prepared with extract and buffer to account for absorbance. The anti-inflammatory activity was expressed as the percentage of BSA denaturation inhibition (Equation 5).

$$\begin{array}{l} \text{I}\left(\text{\%}\right)=\left[\left(\text{Ac}-\text{As}\right)/\text{As}\right]\times 100 \end{array}$$ (5)

where, *I* is the inhibition ratio, A_C_ the control absorbance, and A_S_ the sample absorbance.

### 2.13 *In vivo* anti-inflammatory activity

The *in vivo* anti-inflammatory effect of the extract was evaluated in albino Swiss male mice (20–22 g) obtained from the Pasteur Institute (Algiers). Upon arrival, animals were acclimatized for 1 week under controlled laboratory conditions and housed in polypropylene cages (4–5 mice per cage) with stainless steel covers. Environmental conditions were maintained at 22 ± 2 °C, relative humidity of 50%−60%, and a 12 h light/dark cycle. Animals had free access to standard pellet diet and water *ad libitum*, and environmental enrichment was provided to reduce stress. Mice were randomly divided into three groups (*n* = 5 per group): control (0.5 mL saline), reference (diclofenac sodium, 10.0 mg/kg), and experimental (0.5 mL extract solution, 10.0 w/v %). After 16 h of fasting, treatments were administered by oral gavage. After 30 min, 0.025 mL of 1.0 w/v % carrageenan was injected subcutaneously into the left hind paw to induce inflammation. Ketamine (90 mg/kg, intraperitoneal injection) was administered to minimize pain and distress during the experimental procedures, in accordance with institutional ethical guidelines and ARRIVE guidelines. At the end of the experiment, euthanasia was performed by cervical dislocation, following approved ethical standards for the humane treatment of laboratory animals. All efforts were made to minimize animal suffering throughout the study. The hind limbs were then removed, and edema was assessed by weighing both limbs. The anti-inflammatory activity was evaluated by calculating the percentage reduction in edema (Equation 6) in treated groups compared with the control group:


$$\text{I}(\%)\text{ of edema=}\frac{\left(\text{PG - PD}\right)\text{ control mouse group - }\left(\text{PG - PD}\right)\text{ treated mouse group}}{\left(\text{PG - PD}\right)\text{ control mouse group}}\;\times \text{100}$$

Where PG is the weight of the left hind limb and PD is the weight of the right limb.

### 2.14 *In silico* evaluation of the *M. chamomilla* L. extract phytoconstituents as HER2 and ERα inhibitors

The inhibitory potential of *M. chamomilla* extract phytoconstituents against two key breast cancer targets, human epidermal growth factor receptor 2 (HER2) and estrogen receptor alpha (ERα), was assessed using molecular docking, a computational approach widely employed in drug discovery to model ligand–receptor interactions. The structures of the phytoconstituents identified in the *M. chamomilla* extract and of the reference drugs doxorubicin and genistein were retrieved in 3D SDF format from the PubChem database (http://pubchem.ncbi.nlm.nih.gov). These structures were imported into the molecular operating environment (MOE) software for database creation and molecular preparation. Hydrogen atoms were added, and energy minimization was performed using the MMFF94x force field. The resulting structures were saved in .mdb format for the molecular docking analysis (28). The crystal structures of the target proteins ERα (PDB ID: 3ERT) and HER2 (PDB ID: 7PCD) were retrieved from the Protein Data Bank (https://www.rcsb.org) (29–31). Water molecules and heteroatoms were removed, and hydrogen atoms were added to preserve the structural integrity. Partial charges were assigned, and energy minimization was performed using the AMBER99 force field to optimize protein conformations and resolve steric clashes (32). Docking grids were generated based on the active site coordinates of the co-crystallized ligands. Ligand docking was performed using the Triangular Matcher placement algorithm. Binding affinities were initially scored using the London dG scoring function and then rescored using the WSA/GBVI dG method. For each ligand, 30 poses were generated, and the top five scoring conformations were retained for further analysis.

### 2.15 Statistical analysis

IC50 5 values were determined using linear regression, while variance analysis was performed with ANOVA. Results are presented as mean ± SD from three measurements. The Tukey test was used to identify significant differences between means, with *p*-values < 0.05 considered significant.

## 3 Results and discussion

### 3.1 Chemical composition of the *M. chamomilla* L. extract

The TPC of the *M. chamomilla* L. extract from the semi-arid region of Algeria was 41.90 ± 0.32 mg GAE/g extract. This is higher than the TPC values reported for *M. chamomilla* L. extracts from Morocco (19.62 mg GAE/g) and Iran (7.94 mg GAE/g), but slightly lower than the value from northern Algeria (50.75 mg GAE/g) (Table 1). These differences highlight how environmental conditions and extraction methods can influence the concentration of phenolic compounds. As phenolic compounds are known for their antioxidant effects, the high TPC in the Algerian sample suggests that it retains significant antioxidant potential. The TFC of the Algerian extract was 17.05 ± 1.24 mg RE/g and was similar to the TFC for the *M. chamomilla* L. extract from Morocco (17.2 mg RE/g), but much higher than those from Egypt (1.8 mg RE/g) and Iran (3.15 mg RE/g). This indicates that the *M. chamomilla* L. from the Algerian semi-arid region is relatively rich in flavonoids compared with other regions. Flavonoids have antioxidant and anti-inflammatory properties that can enhance the plant therapeutic value.

**Table 1:** Chemical composition and extraction yield of *M. chamomilla L*. extracts from different areas.

Area	TPC (mg GAE/g extract)	TFC (mg RE/g extract)	Yield (%)	References
Semi-arid region of Algeria	41.90 ± 0.32	17.05 ± 1.24	18.322 ± 0.336	This study
Northern Algeria (humid climate)	50.75	36.72	15.2	(33)
Egypt	22.4	1.8	ND	(34)
Morocco	19.62	17.2	16.76	(35)
Iran	7.94	3.15	ND	(36)

ND, not determined. TPC is expressed as μg gallic acid equivalents/mg of extract (μg GAE/mg). TFC is expressed as μg quercetin equivalents/mg of extract (μg QE/mg). TPC and TFC were calculated by linear regression analysis and expressed as mean ± SD (*n* = 3).

### 3.2 Liquid chromatography-mass spectrometry analysis LC-MS-MS

**Figure 1:**
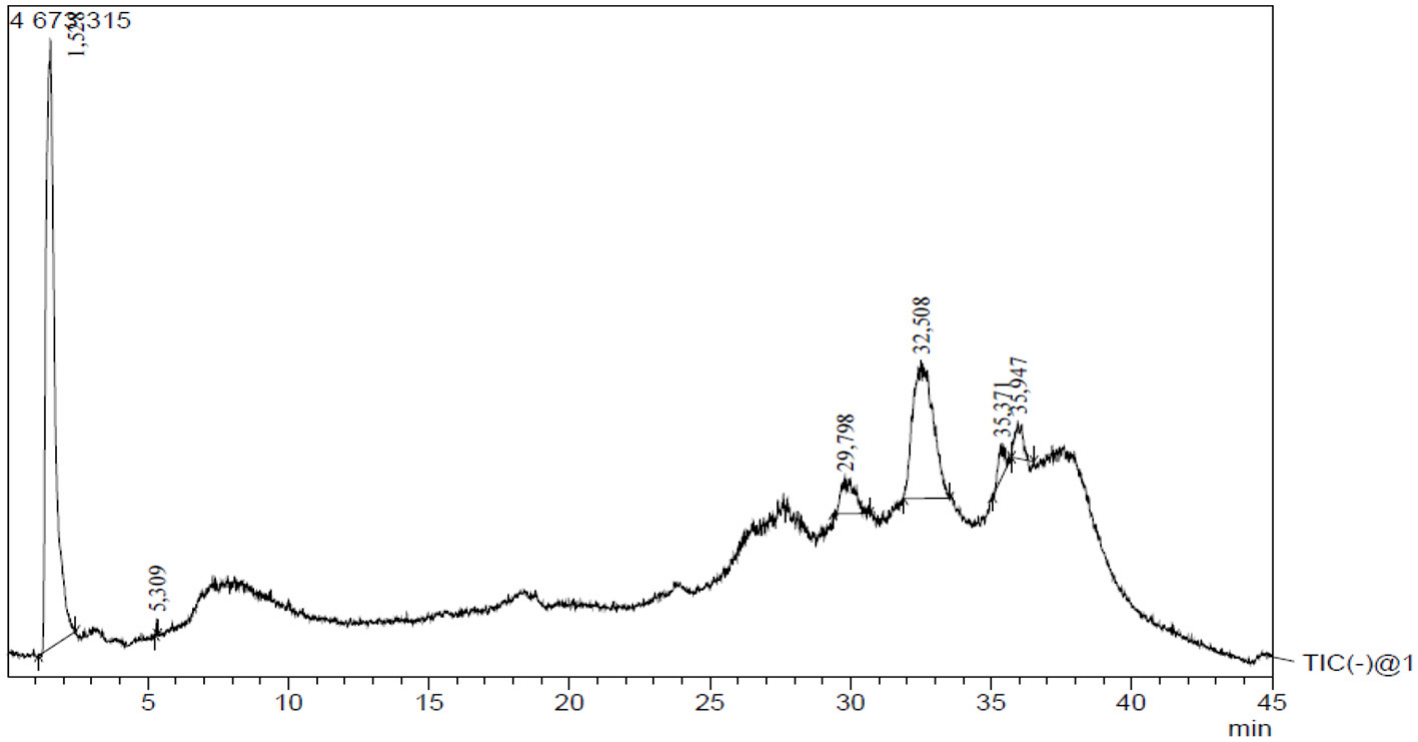
LC-MS/MS profiles of *Matricaria chamomilla L*. extract in negative ion mode (TIC—@1).

**Figure 2:**
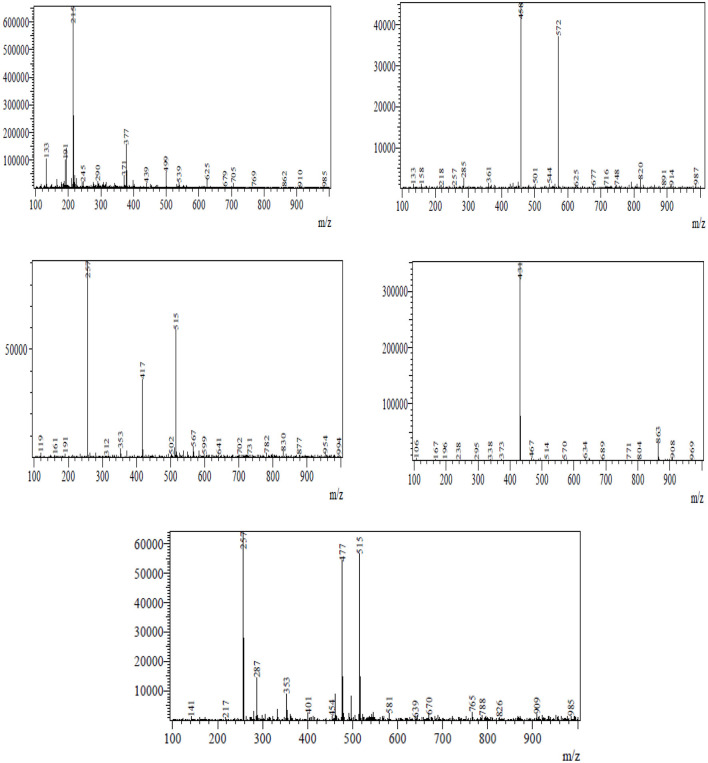
Mass spectra of detected compounds from *Matricaria chamomilla* L. extract showing m/z values versus signal intensity (counts).

**Figure 3:**
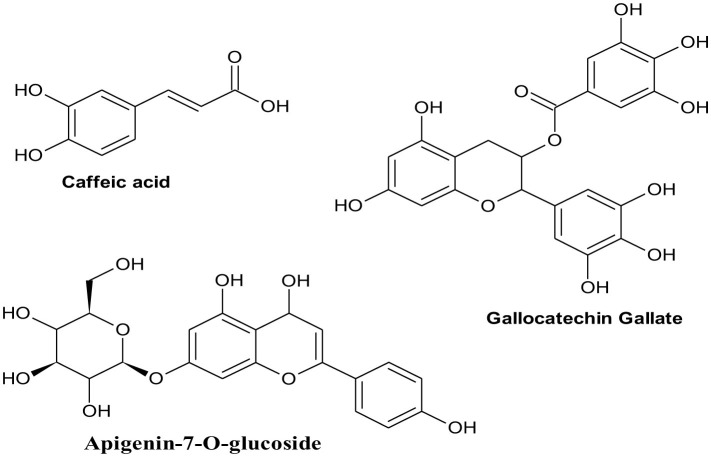
The structure major components detected via LC-MS/MS in *M. chamomilla* L.

After establishing the optimal UPLC-ESI-MS-MS conditions, the *Matricaria chamomilla* L. extract was analyzed utilizing the method's full scan with negative ions mode (see Figure 1 and Table 2). Figures 2, 3 illustrate the UPLC-ESI-MS-MS and mass spectra of eleven chemicals identified from *Matricaria chamomilla* L. extract. Table 3 outlines retention time (Rt), m/z, and the formula of compounds proposed or deduced based on data reported in previously identified Matricaria genus. HPLC-ESI-MS/MS analysis of phenolic compounds from *Matricaria chamomilla* L. revealed a strong variety of secondary metabolites. Molecular analysis reveals that flavonoids and their derivatives constitute the predominant molecular class.

**Table 2:** Phenolic profile determined by LC-MS-MS in fraction from *Matricaria chamomilla* L.

N°	*t*_R_ (min)	IM *(m/z)*	*m/z*	Tentatively identified compound	Molecular formula	Class	References
1	1,528	[M–H]^−^	215	Caffeic acid	C_21_H_20_O_11_	Phenolic acid	(1, 2)
2	5,309	[M–H]^−^	457	Gallocatechin gallate	C_8_H_10_O_9_	Gallate ester	(3)
3	29,798	[M–H]^−^	258	ND	-	-	
4	35,371	[M–H]^−^	431	Apigenin-7-O-glucoside	C_21_H_20_O_10_	Flavone	(4)
5	35,947	[M–H]^−^	257	ND	-	-	

RT, retention time; m/z, ND, not detected.

**Table 3:** *In vitro* antioxidant activity analysis of the *M. chamomilla* L. extract.

Sample	DPPH	GOR	β-Carotene	ABTS
	IC_50_ (μg/mL)	IC_50_ (μg/mL)	IC_50_ (μg/mL)	IC_50_ (μg/mL)
*M. chamomilla* extract	324.4 ± 3.33^a^	22.72 ± 0.08^a^	522.14 ± 0.36^a^	>800
BHT^*^	12.99 ± 0.41^b^	3.32 ± 0.18^b^	0.91 ± 0.01^b^	Nt
BHA^*^	6.14 ± 0.41^c^	5.38 ± 0.06^c^	1.05 ± 0.03^c^	15.21 ± 0.77^a^
Vitamin E^*^	20.81 ± 0.64^d^	Nt	Nt	37.79 ± 0.28^a^

Values are the mean ± SD of three parallel measurements.

^*^Standard compounds. NT, not tested. IC_50_ are the concentrations corresponding to 50% inhibition. Values marked with different superscripts (a, b, c, d, e, f, g, h) within the same columns differ significantly (*p* < 0.05).

Three principal compounds were identified within this family, which comprises the following: gallocatechin gallate was preliminarily identified in this species for the first time. Gallocatechin gallate has been confirmed to demonstrate a wide range of biological activities (5). Gallocatechin gallate has shown efficacy in lowering blood glucose levels and protecting pancreatic β cells in diabetic rats. The hypoglycemic effects of gallocatechin gallate, a primary component of crude tea leaves from *Camellia sinensis*, were examined in streptozotocin-induced diabetic rats (6). Salmerón-Manzano et al. reported that administration of gallocatechin gallate significantly protected HT22 cells from H_2_O_2_-induced cytotoxicity by suppressing and alleviating the formation of reactive oxygen species in different amounts. Furthermore, radical scavenging activity was observed in all analyzed catechins, which partially eliminated free radicals (7). Epigallocatechin gallate surpassed all other catechins in reactive oxygen species generation and radical scavenging activity, elucidating the results presented in this paper and suggesting that gallocatechin gallate may account for the remarkable effect.

Caffeic acid was also detected and has been previously reported in *Matricaria chamomilla*, although it is here highlighted within the identified phytochemical profile (3). Apigenin-7-O-glucoside previously reported in other Matricaria species (8–10). The findings of Wei Wang et al. indicate the potential of apigenin-7-O-glucoside, a principal component identified in the studied species (9), as a pharmacological agent for antioxidation and anti-inflammation, with the concomitant use of AG and trolox potentially enhancing its efficacy. Our findings will yield novel insights into the creation of pharmaceuticals with antioxidative and anti-inflammatory properties (11). Additionally, Esen et al. demonstrated that apigenin-7-O-glucoside has an antibacterial action, and their findings demonstrated that it was effective against Enterococcus faecalis, Klebsiella pneumoniae, and Staphylococcus aureus (12).

The presence of caffeic acid has been reported in other studies (10, 13, 14) as the main phenolics in *Matricaria chamomilla*. Diabetic rats treated with caffeic acid demonstrate favorable effects across all metabolic markers. Moreover, caffeic acid exhibits considerable ABTS radical scavenging, DPPH radical dot scavenging, superoxide anion radical scavenging, total reduction power, and ferrous ion chelation activities, as analyzed in this paper based on prior studies. Consequently, we can ascribe this significant antioxidant activity to the presence of caffeic acid, which is recognized as an antioxidant agent. In this study, caffeic acid shown a positive impact and antidiabetic properties in comparison to individual treatments (15), which explain the strong effect of *Matricaria chamomilla* across the alpha amylase enzyme. The findings of Fernanda et al. corroborate and expand upon existing literature, demonstrating that caffeic acid derivatives, identified through LC-MS/MS, exhibit both *in vitro* and *in vivo* anti-inflammatory effects. These effects are mediated, at least in part, by the scavenging of nitric oxide and their capacity to modulate iNOS expression, as well as potentially influencing other inflammatory mediators (16). Furthermore, caffeic acid demonstrates significant ABTS radical scavenging, DPPH radical dot scavenging, superoxide anion radical scavenging, total reducing power, and ferrous ion chelation activities in earlier studies, as analyzed in this paper. Thus, we can attribute this notable antioxidant activity to the presence of caffeic acid, which is regarded as an antioxidant agent (17).

Overall, our findings are consistent with previous reports, particularly the study by Kim et al., which identified apigenin-7-O-glucoside as a dominant compound in *Matricaria chamomilla* extracts (8). These results further support the rich phytochemical composition and biological potential of this species.

### 3.3 Antioxidant activity

The antioxidant activity of the *M. chamomilla* L. extract was assessed using the DPPH, GOR, β-carotene, and ABTS assays (Table 3) that measure the capacity to scavenge free radicals and inhibit oxidation.

Contrary to the enzyme inhibition results, the antioxidant capacity of the extract was moderate. The DPPH radical scavenging assay showed an IC_50_ value of 324.4 ± 3.33 μg/mL, which is significantly higher (and less potent) than the synthetic antioxidant BHT (IC_50_ = 12.99 ± 0.41 μg/ml). This difference confirms that although the extract is a potent enzyme inhibitor, its primary mechanism of action in this study may not be through direct free radical scavenging. Similarly, the IC_50_ value obtained with the GOR assay (22.72 ± 0.08 μg/mL) showed that the extract was moderately effective in stabilizing free radicals compared with BHT (3.32 ± 0.18 μg/mL) and BHA (5.38 ± 0.06 μg/mL). In the β-carotene bleaching assay, the extract inhibited lipid peroxidation very weakly compared with BHT and BHA (IC_50_ = 522.14 ± 0.36 μg/mL vs. 0.91 ± 0.01 μg/mL and 1.05 ± 0.03 μg/mL, respectively). Lastly, the absence of measurable results for the ABTS assay suggested the lack of or minimal antioxidant activity of the extract in this context. In conclusion, the *M. chamomilla* L. extract antioxidant capacity, especially in the DPPH and GOR assays, was good but much lower than that of the synthetic antioxidants BHT and BHA. Nonetheless, because of the presence of phenolic compounds, the *M. chamomilla* L. extract could be an interesting natural alternative for health supplements or food preservation. Nonetheless, the oxidative tests demonstrate rather weak antioxidant action in *M. chamomilla* L., occasionally ascribed to a “lack of synergy” (or even antagonism). Plant extracts constitute complex combinations. Compounds may engage in chemical interactions (e.g., binding, oxidation, or competing for radicals) or mitigate each other's effects. This is how to elucidate this seeming conflict. *M. chamomilla* L. is abundant in bioactive chemicals, including flavonoids (quercetin, apigenin, luteolin), phenolic acids (caffeic, chlorogenic, ferulic), coumarins, and terpenoids. These factors enhance its repute as a source of antioxidants in several studies. Chamomile continues to be valued for its antioxidant properties, however outcomes differ. Optimized extracts (e.g., ethanol or subcritical water) or their mixtures with additional antioxidants frequently demonstrate superior efficacy compared to crude teas or infusions in certain experiments.

### 3.4 *In vitro* antimicrobial activity

The antimicrobial effects of the *M. chamomilla L*. extract were assessed with the disk diffusion method and different microorganisms (Table 4).

**Table 4:** Antimicrobial activity of the *M. chamomilla L*. extract against different bacterial and fungal strains: inhibition zones.

Strain		Extract	References used	
Gram(+) bacteria	*S. aureus* ATCC 6538	22.0 ± 0.8^a^	Fosfomycin 44.0 ± 0.5^b^	Carbenicillin 37.5 ± 0.4^c^
Gram(+) bacteria	*B. subtilis* ATCC 6633	12.0 ± 0.8^a^	Erythromycin 32.5 ± 0.3^b^	Cephalexin 31.0 ± 0.3^c^
Gram(–) bacteria	*P. aeruginosa* ATCC 9027	13.5 ± 0.8^a^	Fosfomycin 31.2 ± 0.2^b^	NT
Gram(–) bacteria	*E. coli* ATCC 8739	12.0 ± 0.8^a^	Fosfomycin 33 ± 0.3^b^	NT
Fungal strain	*C. albicans* ATCC 10231	18.0 ± 0.8^a^	NT	NT

^*^Standard compounds. NT, not tested. The values expressed as mean ± SD (*n* = 3). Values marked with different superscripts (a, b, c) within the same lines differ significantly (*p* < 0.05).

The MIC values of plant extracts and pure phenolic acids were assessed to evaluate their antibacterial efficacy against certain foodborne pathogenic bacteria. The MIC method is better suitable for quantitatively assessing antibacterial activity, as it demonstrates the efficacy of plant extracts at lower concentrations compared to the disc diffusion approach. The MIC values for Matricaria chamomilla extract in this study ranged from 0.5 to 6.25 μg/mL, signifying moderate antibacterial activity based on established criteria for natural products. On the other hand, it is not appropriate to make a direct comparison with synthetic antibiotics because of the fundamental differences in the chemical complexity and potency of the two. While bioactive compounds often exhibit lower MICs, the crude extract may benefit from synergistic interactions among phenolics, flavonoids, and terpenoids, as evidenced by. These multi-target mechanisms may improve efficacy *in vivo*, notwithstanding comparatively elevated *in vitro* MICs.

The extract displayed notable antibacterial effects against Gram-positive bacteria, as indicated by the inhibition zones of 22.0 ± 0.8 mm against *S. aureus* and of 12 ± 0.75 mm for *B. subtilis*. In both cases, it was less effective than fosfomycin (44.0 ± 0.5 mm) and carbenicillin (37.5 ± 0.4 mm), and of erythromycin (32.5 ± 0.3 mm) and cephalexin (31 ± 0.3 mm), respectively. The extract was also moderately effective against the Gram-negative bacteria *E. coli* and *P. aeruginosa* (inhibition zones of 13.5 ± 0.8 mm and 12.0 ± 0.8 mm), but less than fosfomycin (31.2 ± 0.2 mm and 33.0 ± 0.3 mm, respectively). The extract also significantly inhibited *C. albicans* (inhibition zone of 18.0 ± 0.8 mm), potentially due to its phenolic compounds. No reference antifungal agent was used for comparison. The antimicrobial effects of the *M. chamomilla L*. extract were investigated also by quantifying its MIC against different fungal and bacterial strains (Table 5).

**Table 5:** Minimum inhibitory concentration values (μl/mL) of the *M. chamomilla L*. extract.

Strain	Extract (μl/mL)	Positive control
**Gram(+) bacteria**		
*L. monocytogenes* CIP82110	6.125	Levofloxacin (0.125)
*B. subtilis* ATCC 6633	6.125	Levofloxacin (0.125)
*S. aureus* ATCC 6538	0.500	Levofloxacin (0.062)
**Gram(–) bacteria**		
*E. coli* ATCC 8739	1.530	Levofloxacin (0.062)
*K. pneumoniae* CIP 8291	6.125	Levofloxacin (0.125)
*P. aeruginosa* ATCC 9027	< 0.760	Levofloxacin (1)
Fungi		
*M. ramannianus*	6.125	Nystatin (0.94)
*A. flavus*	6.125	Nystatin (0.94)
*P. expansum*	1.530	Nystatin (0.23)
*F. culmorum*	< 0.760	Nystatin (0.007)

The MIC results indicated that the extract had low activity against *L. monocytogenes* and *B. subtilis* (6.125 μl/mL), and moderate activity against *S. aureus* (0.5 μl/mL) compared with the positive control levofloxacin (MIC values from 0.062 to 0.125 μl/mL). The extract was particularly effective against *P. aeruginosa* (MIC < 0.76 μl/mL), but less against *E. coli* (MIC = 1.53 μl/mL) and *K. pneumoniae* (MIC = 6.125 μl/mL). Among the tested fungal strains, the lowest MIC with the extract was obtained against *F. culmorum* (< 0.76 μl/mL). Nystatin exhibited superior antifungal efficacy, especially against *F. culmorum* (0.007 μl/mL) and *P. expansum* (0.23 μl/mL). These results highlight the potential of *M. chamomilla* as a natural antimicrobial agent, although it is generally less potent than conventional antibiotics and antifungals.

### 3.5 *In vitro* antidiabetic activity

**Figure 4:**
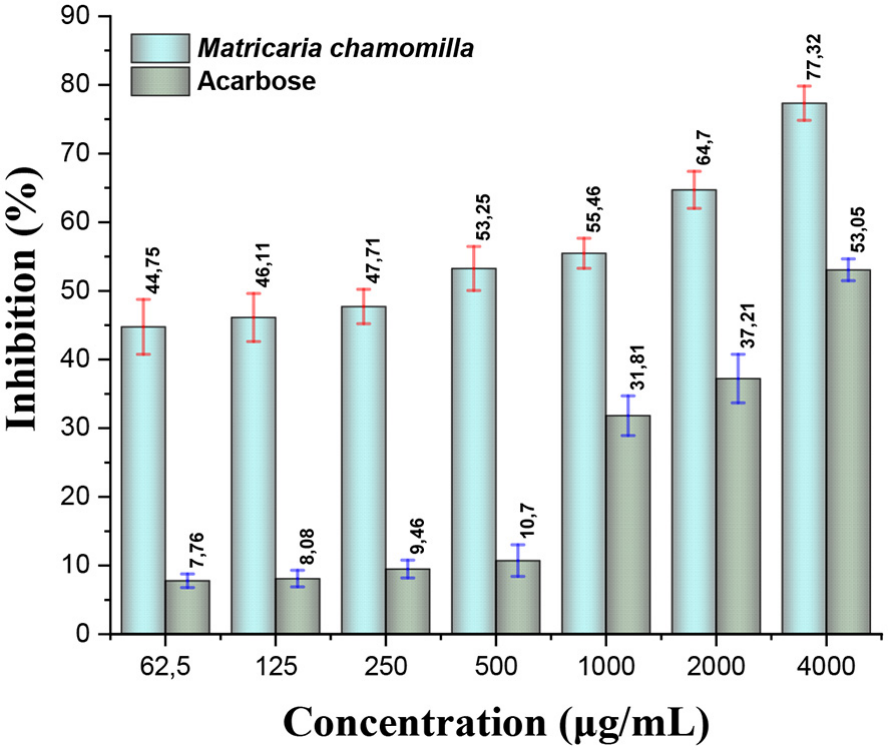
*In vitro* antidiabetic activity (percentage of α-amylase inhibition) of the *M. chamomilla* extract and of acarbose (standard inhibitor) at different concentrations.

The antidiabetic potential of the *M. chamomilla L*. extract was assessed with the α-amylase inhibition assay (Figure 4).

The aqueous methanolic extract of chamomile exhibited a significant inhibitory effect on alpha-amylase, with an IC_50_ value of 440.6 μg/mL. Under the same experimental conditions, the commercial inhibitor acarbose showed an IC_50_ value of 3650.9 μg/mL. While these results suggest a strong inhibitory effect of the crude extract, it is important to note that the extract's efficacy may stem from the synergistic interaction of its phytochemical components, such as caffeic acid and apigenin-7-O-glucoside, rather than from a single biological agent. This highlights *M. chamomilla* extract as a more effective natural alternative for controlling blood sugar levels (37). Its enhanced inhibitory effect could be attributed to its rich phytochemical composition, particularly flavonoids, tannins and saponins. Gowri et al. (38) and Drouin et al. (37) identified flavonoids as potent α-amylase inhibitors. Moreover, the extract inhibited α-amylase activity in a concentration-dependent manner: from 44.75 ± 0.11% at 62.5 μg/mL to 77.32 ± 0.11% at 4,000 μg/mL. Conversely, acarbose showed lower inhibition across all concentrations, with a maximum of 53.05 ± 1.59% at 4,000 μg/mL. These findings underscore the potent antidiabetic effect of the *M. chamomilla* extract (*P* < 0.001 vs. acarbose), particularly at lower concentrations, suggesting that it may offer a more effective and potentially safer method for controlling blood glucose levels compared with synthetic inhibitors (39).

### 3.6 *In vitro* anticholinesterase activity

**Figure 5:**
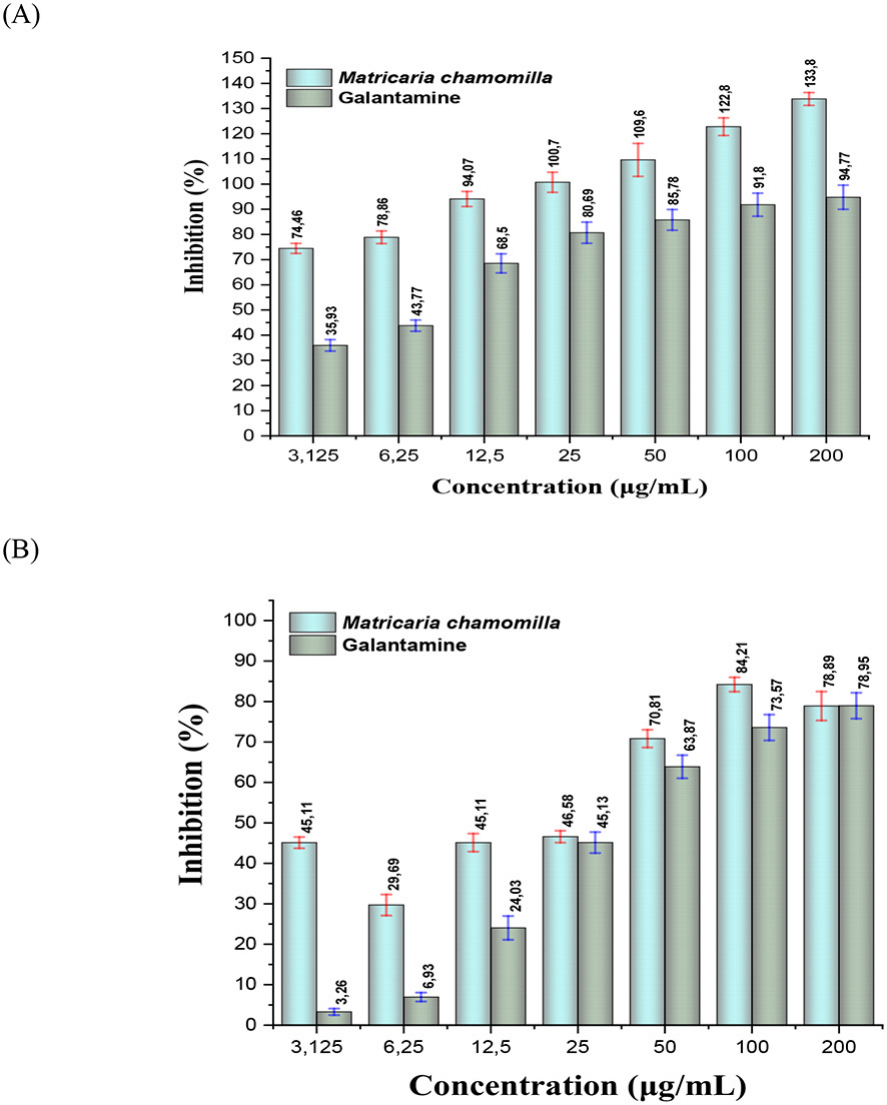
*In vitro* anticholinesterase activity of the *M. chamomilla* L. extract: inhibition of acetylcholinesterase activity **(A)** and of butyrylcholinesterase activity **(B)** compared with galantamine (reference standard).

The *in vitro* anticholinesterase activity of the *M. chamomilla* L. extract was evaluated by assessing its inhibitory effects on BChE and AChE, compared with galantamine (reference standard), using the Ellman's method as described in Ghribia et al. (26) (Figure 5).

In an acetylcholinesterase inhibition assay, the extract demonstrated significant activity with an IC_50_ value of 3.11 μg/mL. This value is numerically lower than that recorded for the reference drug galantamine (IC_50_ = 6.27 μg/mL) tested under similar conditions. These results suggest that semi-dried Algerian chamomile possesses potent neuroprotective properties. However, further biologically targeted fractionation is needed to isolate the specific compounds responsible for this strong inhibition and to validate these preliminary observations *in vitro* (Figure 5A). As well as the extract also displayed good BChE inhibition (IC_50_ value of 28.69 ± 2.29 μg/mL vs. 34.75 ± 1.98 μg/mL for galantamine; Figure 5B). The difference between the *M. chamomilla* L. extract and galantamine was significant in both cases (*P* < 0.05). The pronounced inhibitory effects observed against AChE and BChE underscore the therapeutic potential of *M. chamomilla* for Alzheimer's disease treatment by targeting enzymes implicated in cognitive decline. This high efficacy is likely due to the presence of polyphenols in the extract that have known neuroprotective properties. For instance, Dastmalchi et al. (40) highlighted the significant role of phenolic compounds in neuroprotection, particularly from plants of the *Lamiaceae* family, including *M. chamomilla*. Vladimir-Knedimir et al. (41) reported strong AChE inhibition by *M. chamomilla* extracts, particularly from plants grown in Croatia. Collectively, these findings emphasize that *M. chamomilla* extracts are a promising candidate for developing new treatments for Alzheimer's disease by effectively inhibiting key enzymes implicated in neurodegeneration.

### 3.7 *In vitro* anti-inflammatory activity

**Figure 6:**
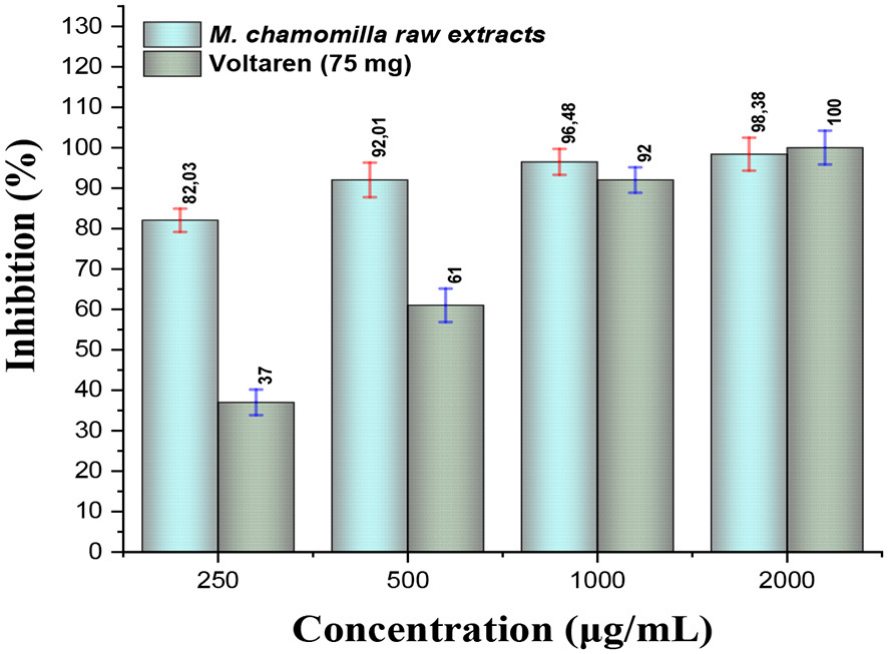
*In vitro* anti-inflammatory activity (inhibition of BSA denaturation) of the *M. chamomilla* L. extract compared with diclofenac sodium (standard anti-inflammatory).

The *in vitro* anti-inflammatory activity of the *M. chamomilla* L. extract was assessed by measuring the inhibition of BSA denaturation, a marker of inflammation (Figure 6), at various concentrations (250–2,000 μg/mL), compared to diclofenac sodium, a standard anti-inflammatory drug.

BSA denaturation inhibition by the extract ranged from 82.03% at 250 μg/mL to 98.38% at 2,000 μg/mL, approaching the 100% inhibition observed with 75 mg of diclofenac sodium. This significant suppression may be ascribed to the phenolic and flavonoid components in the extract, recognized for their anti-inflammatory characteristics. The concentration-dependent inhibitory effect suggests that the *M. chamomilla* extract could serve as a valuable therapeutic agent for inflammatory conditions, potentially providing an alternative or complementary option to conventional anti-inflammatory drugs (42, 43).

### 3.8 Acute toxicity

The extract of *Matricaria chamomilla* exhibited a high degree of biological safety, as neither mortality nor notable behavioral and clinical abnormalities were observed in mice, even at the maximum dosage of 2,000 mg/kg. No signs of toxicity, such as hyperactivity, ataxia, convulsions, or lethargy, were observed. These findings further substantiate the extract's potential as a safe natural source of anti-inflammatory and analgesic compounds. Furthermore, they advocate for the progression of plant-based substitutes for traditional pharmacological treatments, which are often limited by detrimental toxicity.

### 3.9 *In vivo* anti-inflammatory activity

The *in vivo* anti-inflammatory activity of the *M. chamomilla* L. extract was assessed using a mouse model of carrageenan-induced inflammation in the left hind paw. The results indicated a reduction in paw edema, although less effectively than the standard drug diclofenac sodium (–38% vs. –53.0%; Table 6). The observed anti-inflammatory effect of the extract could be attributed to its high polyphenolic and flavonoid content, as indicated by the TPC of 41.90 mg GAE/g and TFC of 17.05 mg RE/g. These compounds are recognized for their strong antioxidant and anti-inflammatory characteristics, which contribute to the extract therapeutic potential.

**Table 6:** *In vivo* anti-inflammatory activity of the *M. chamomilla* L. extract in a classical inflammation mouse model (carrageenan-induced edema in the left hind paw).

Treatment groups (*n* = 5 mice/group)	Mean hind paw weight (g)		% Edema	% Edema reduction
	Left	Right		
Negative control (saline solution)	0.109 ± 0.007^a^	0.076 ± 0.005^a^	43.4%	0.00%
*M. chamomilla* (aqueous extract)	0.181 ± 0.004^b^	0.143 ± 0.002^b^	26.6%	38.8%
Reference (diclofenac sodium)	0.171 ± 0.008^c^	0.142 ± 0.002^c^	20.4%	53.0%

^*^Standard compounds. NT, not tested. The values expressed as mean ± SD (*n* = 5). Values marked with different superscripts (a, b, c) within the columns lines differ significantly (*p* < 0.05).

Although diclofenac sodium was more potent in reducing inflammation, the *M. chamomilla* extract offers several advantages as a natural alternative. Its polyphenolic and flavonoid components target inflammation and also provide antioxidant benefits, unlike diclofenac. Additionally, *M. chamomilla* has broader therapeutic properties, including antimicrobial, antidiabetic and anticholinergic effects, making it a multifunctional bioactive agent. The extract lower risk of adverse side effects, particularly gastrointestinal and cardiovascular adverse events, highlights its potential as a safer alternative. Moreover, it may be useful in combination therapy to reduce the required dose of conventional drugs, minimizing the associated risks while retaining anti-inflammatory efficacy.

### 3.10 *In silico* evaluation of the *M. chamomilla* L. extract phytoconstituents as HER2 and ERα inhibitors

To explore the potential interactions of M. chamomilla phytoconstituents with breast cancer-related molecular targets, molecular docking simulations were conducted against ERα (PDB ID: 3ERT) and HER2 (PDB ID: 7PCD). The docking protocol was first validated through redocking of the native co-crystallized ligands (44, 45), yielding RMSD values of 0.585 Å for ERα and 0.734 Å for HER2, both within the acceptable threshold (< 2.0 Å), thus supporting the reliability of the docking setup (Table 7) (46).

**Table 7:** Redocking of co-crystallized ligands into the active sites of the studied target proteins.

Protein targets	PDB ID	Co-crystalized ligand	Conformation	Binding energy (kcal/mol)	RMSD (Å)
Erα	3ERT	4-Hydroxytamoxifen (OHT)	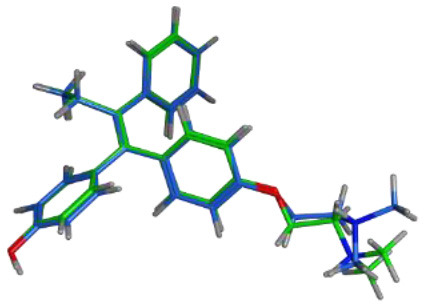	–9.6844	0.585
HER2	7PCD	1-[4-[4-[[3,5-Bis(chloranyl)-4- ([1,2,4]triazolo[1,5-*a*]pyridin-7- yloxy)phenyl]amino]pyrimido[5,4- *d*]pyrimidin-6-yl]piperazin-1-yl]-4-(3- fluoranylazetidin-1-yl)butan-1-one	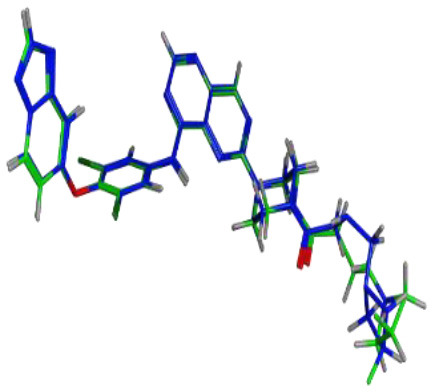	–12.1018	0.734

ERα, human estrogen receptor alpha; HER2, human epidermal growth factor receptor 2. The blue color represents the conformation of the native co-crystallized ligand, and the green color represents the conformation obtained after redocking.

Among the investigated compounds, α-tocopherol exhibited the lowest docking energy values toward both ERα (−8.78 kcal/mol) and HER2 (−8.68 kcal/mol; Table 7), indicating a relatively strong binding affinity within the context of this computational model. These values were lower than those obtained for the reference compounds genistein and doxorubicin, suggesting a potentially favorable interaction profile. However, it should be emphasized that such comparisons remain limited to theoretical predictions and do not necessarily reflect actual biological efficacy.

Similarly, luteolin 7-glucoside and apigenin 7-glucoside demonstrated notable binding affinities, particularly toward HER2, while hesperidin showed moderate interaction with ERα. Other flavonoids, including isorhamnetin, acacetin, chrysin, and penta-hydroxyflavone, displayed comparatively weaker binding energies, suggesting a lower predicted affinity for the studied targets. The reference compounds genistein and doxorubicin showed moderate docking scores under the same conditions. While some phytoconstituents exhibited more favorable binding energies *in silico*, these findings should be interpreted with caution, as docking scores alone are not sufficient to establish pharmacological superiority or therapeutic relevance. At the molecular level, α-tocopherol interactions were primarily stabilized through hydrophobic contacts with key residues within the binding pockets of both receptors. This behavior is consistent with its lipophilic structure and may contribute to its favorable docking energy. Likewise, apigenin 7-glucoside formed both hydrogen bonds and hydrophobic interactions, which may enhance binding stability within the simulated environment (47, 48).

**Figure 7:**
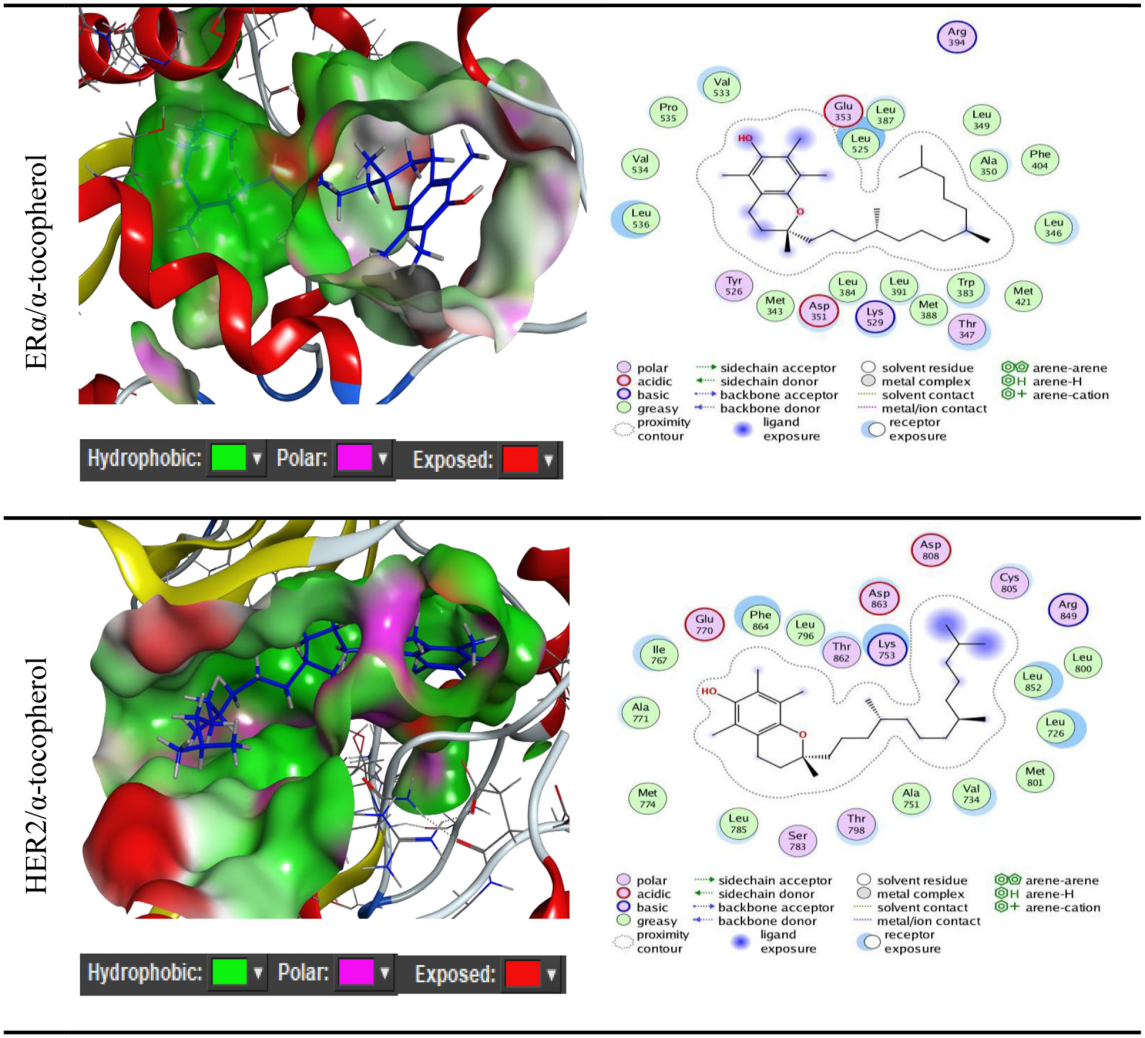
2D and 3D molecular docking simulation studies of the interactions between α-tocopherol and the active sites of ERα (PDB ID: 3ERT) and HER2 (PDB ID: 7PCD).

Among the tested phytochemicals, α-tocopherol demonstrated the highest binding affinities toward both ERα and HER2, with docking scores of −8.78 and −8.68 kcal/mol, respectively (see Table 8). These values surpass those of both standard anticancer agents genistein and doxorubicin suggesting that α-tocopherol strongly and stably interacts with both targets (28). Notably, α-tocopherol exclusively mediated hydrophobic interactions without hydrogen bonds observed (Figure 7). This lack of hydrogen bonds is likely due to its predominantly hydrophobic structure and the absence of polar functional groups. In the binding pocket of ERα, hydrophobic stabilization was provided by the Val533, Val534, Pro535, Leu536, Leu525, Leu387, Leu349, Leu346, Leu391, Leu384, Met343, Met388, Met421, Ala350, Trp383, and Phe404 residues. Likewise, in HER2, Phe864, Leu726, Leu785, Leu796, Leu800, Leu852, Met774, Met801, Val734, Ala751, Ala771, and Ile767 contributed to the hydrophobic interactions that stabilized the complex.

**Table 8:** Binding energy (kcal/mol) of *M. chamomilla* compounds and reference drugs.

ID PubChem	Compounds	Binding energy (kcal/mol)	
		ERα	HER2
14985	Alpha-tocopherol	–8.7773	–8.6775
5280637	Luteolin 7-glucoside	–6.9584	–8.0783
10621	Hesperidin	–7.5056	–6.7510
5280704	Apigenin 7-glucoside	–7.1267	–8.0911
4114	Xanthotoxin	–5.5966	–6.1168
5281654	Isorhamnetin	–5.4209	–7.1291
90657623	Penta-hydroxyflavone	–5.3800	–6.7790
5280442	Acacetin	–5.9506	–6.8697
5281607	Chrysin	–5.1639	–6.3752
31703	Doxorubicin^*^	–5.7158	–5.7626
5280961	Genistein^*^	–6.8324	–6.4868

ERα, human estrogen receptor alpha; HER2, human epidermal growth factor receptor 2; ^*^, reference drugs.

From a structural perspective, α-tocopherol lipid-soluble chromanol ring coupled with its long phytyl tail enables its deep insertion into the hydrophobic core of both ERα and HER2 binding sites. This exceptional structural complementarity compensates for the absence of hydrogen bonds and contributes to the overall binding stability. The data suggest that α-tocopherol binding mode is uniquely suited for occupying hydrophobic regions, supporting its strong interaction energies and highlighting its potential as a lipophilic inhibitor that targets both ERα and HER2 (49).

**Figure 8:**
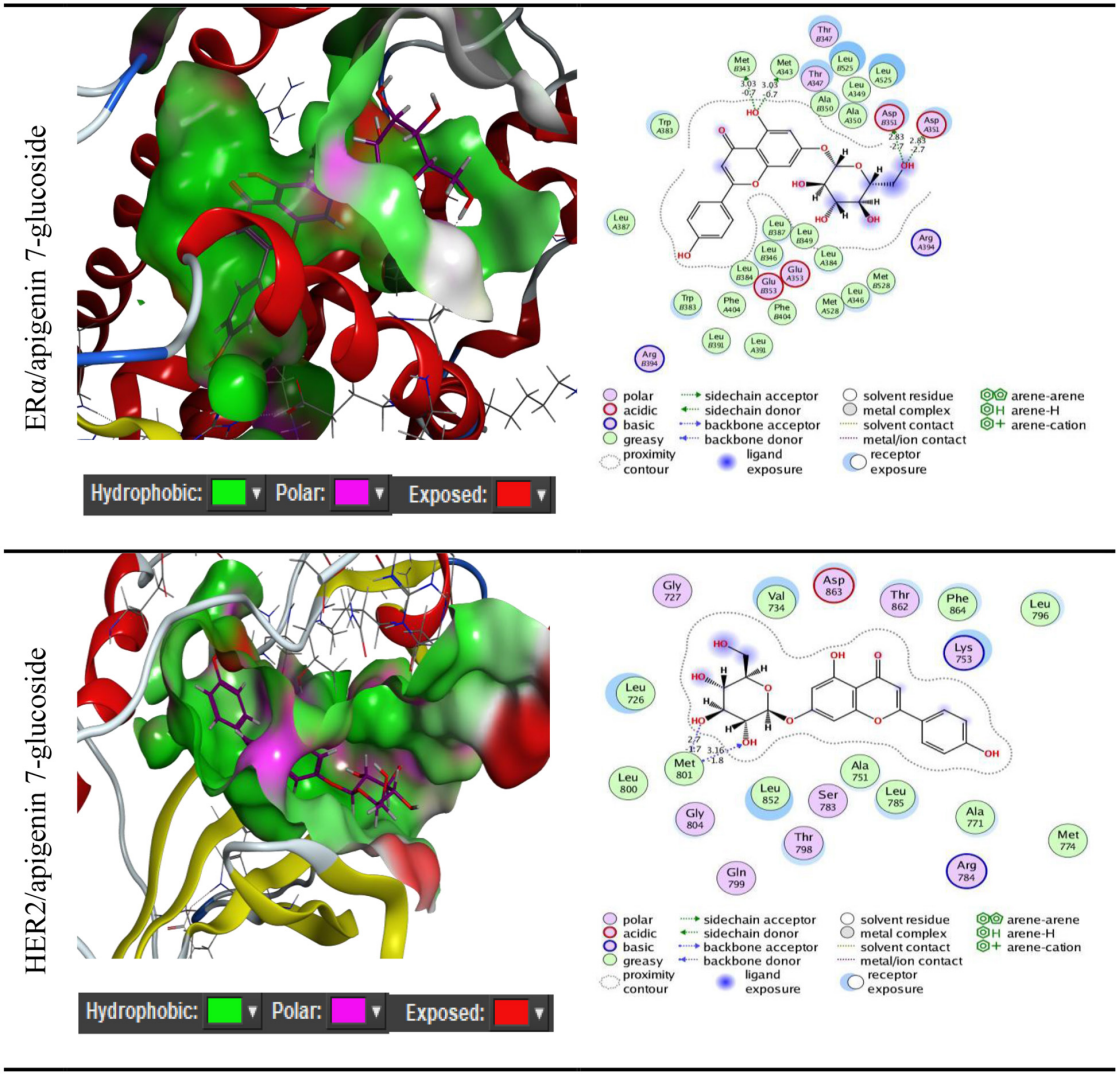
2D and 3D molecular docking simulation studies of the interactions between apigenin 7-glucoside and the active sites of ERα (PDB ID: 3ERT) and HER2 (PDB ID: 7PCD).

The flavonoid apigenin 7-glucoside also exhibited promising inhibitory potential against both ERα and HER2 (binding energies: −7.13 kcal/mol and −8.09 kcal/mol, respectively), indicating strong binding interactions with both targets. These docking scores place apigenin 7-glucoside among the compounds with the highest binding affinities, just below α-tocopherol. Apigenin 7-glucoside formed a network of stabilizing interactions within the active sites of both receptors (Figure 8). In ERα, it established four hydrogen bonds with the residues Met343 and Asp351, and simultaneously engaged in multiple hydrophobic contacts that further enhanced the ligand-receptor complex stability. This combination of polar and non-polar interactions contributes significantly to its strong binding and specificity.

With HER2, apigenin 7-glucoside adopted a well-fitted orientation within the binding pocket where it formed two hydrogen bonds with Met801. These interactions were complemented by surrounding hydrophobic contact with nearby residues, as observed in both the 2D and 3D interaction maps. This dual mode of binding (hydrogen bonding and hydrophobic interactions) suggests that apigenin 7-glucoside can maintain a stable conformation within the receptor cavities. The presence of sugar moiety at position 7 of the apigenin structure likely enhances its interaction with polar residues, while the flavonoid core facilitates π-π and hydrophobic stacking. Altogether, these molecular insights support the potential of apigenin 7-glucoside as a lead compound for targeting ERα and HER2 for breast cancer management.

Molecular docking analysis revealed that alpha-tocopherol and apigenin 7-glucoside are the most potent dual inhibitors of ERα and HER2, suggesting high therapeutic potential for various breast cancer subtypes. In contrast, luteolin 7-glucoside and hesperidin showed moderate affinity due to steric hindrance from bulky sugar moieties, while acacetin, chrysin, and xanthotoxin exhibited the weakest binding. These variations are driven by differences in molecular size and hydrogen-bonding capacity. Overall, while *M. chamomilla* phytoconstituents are promising drug candidates, their predicted efficacy must be validated through *in vitro* and *in vivo* studies to confirm clinical relevance.

Given the critical oncogenic roles of ERα in estrogen-dependent breast cancer and HER2 in HER2-positive subtypes, compounds capable of dual inhibition, such as α-tocopherol and apigenin 7-glucoside, are particularly interesting. Their ability to interact with both targets suggests a broad-spectrum therapeutic potential, possibly offering enhanced efficacy through synergistic or combinatorial strategies. Collectively, the *in silico* molecular docking results emphasize the potential of *M. chamomilla* phytoconstituents as candidates for breast cancer drug development. However, these computational findings must be validated in comprehensive *in vitro* and *in vivo* studies to confirm the stability and biological relevance of the predicted interactions and to assess their clinical transferability.

## 4 Conclusion

This study underscores the significant therapeutic potential of a *M. chamomilla* L. extract from the semi-arid region of Algeria, focusing on its antioxidant, antimicrobial, antidiabetic, anti-inflammatory and neuroprotective properties. The extract high TPC (41.90 mg GAE/g) and TFC (17.05 mg RE/g) correlated with its strong antioxidant activity. The IC_50_ values of 324.4 μg/mL and 22.72 μg/mL obtained with the DPPH and GOR free radical scavenging assays, respectively, confirmed its ability to mitigate oxidative stress, a key factor in the progression of various chronic diseases, including cancer and neurodegenerative disorders. The LC-MS/MS analysis identified several bioactive compounds, particularly caffeic acid, gallocatechin gallate and apigenin-7-O-glucoside. These compounds contribute to the extract diverse pharmacological properties and highlight its therapeutic potential in treating oxidative stress-related conditions. The extract exhibited good antimicrobial activity, as indicated by inhibition zones of 22 mm against *S. aureus* and 18 mm against *C. albicans*. Although it was less effective than the reference antibiotics and antifungals, it still holds promise as a natural antimicrobial agent. Furthermore, its potent α-amylase inhibition (IC_50_: 440.6 μg/mL vs. 3650.9 μg/mL for acarbose) suggests its potential for managing diabetes, particularly in controlling the postprandial blood glucose levels. The extract also inhibited AChE and BChE (IC_50_ = 3.11 μg/mL and 28.69 μg/mL, respectively), outperforming galantamine. This positions it as a potential candidate for the treatment of neurodegenerative disorders, such as Alzheimer's disease. *In vitro*, the extract demonstrated significant anti-inflammatory effects, inhibiting BSA denaturation (98.4% at 2,000 μg/mL) to levels similar to what observed with the reference anti-inflammatory drug diclofenac sodium. *In vivo*, it reduced carrageenan-induced paw edema by 38.8%, further validating its anti-inflammatory properties. *In silico* molecular docking modeling further supported the therapeutic potential of this *M. chamomilla* L. extract by showing that key bioactive compounds (α-tocopherol and apigenin 7-glucoside) bind effectively to ERα and HER2 (two breast cancer targets), underscoring its multifunctional therapeutic promise. These findings warrant further research into the clinical efficacy and mechanism of action of *M. chamomilla* L. for various therapeutic applications.

## Data Availability

The original contributions presented in the study are included in the article/supplementary material, further inquiries can be directed to the corresponding author.
